# Ewing's Sarcoma: Development of RNA Interference-Based Therapy for Advanced Disease

**DOI:** 10.5402/2012/247657

**Published:** 2012-03-11

**Authors:** Olivia Simmons, Phillip B. Maples, Neil Senzer, John Nemunaitis

**Affiliations:** ^1^Gradalis, Inc., Dallas, TX 75201, USA; ^2^Mary Crowley Cancer Research Centers, Dallas, TX 75201, USA; ^3^Texas Oncology, PA, Dallas, TX 75251, USA; ^4^Medical City Dallas Hospital, Dallas, TX 75230, USA

## Abstract

Ewing's sarcoma tumors are associated with chromosomal translocation between the EWS gene and the ETS transcription factor gene. These unique target sequences provide opportunity for RNA interference(i)-based therapy. A summary of RNAi mechanism and therapeutically designed products including siRNA, shRNA and bi-shRNA are described. Comparison is made between each of these approaches. Systemic RNAi-based therapy, however, requires protected delivery to the Ewing's sarcoma tumor site for activity. Delivery systems which have been most effective in preclinical and clinical testing are reviewed, followed by preclinical assessment of various silencing strategies with demonstration of effectiveness to EWS/FLI-1 target sequences. It is concluded that RNAi-based therapeutics may have testable and achievable activity in management of Ewing's sarcoma.

## 1. Introduction

The Ewing's sarcoma family of tumors (ESFT) are a group of solid bone malignancies most commonly occurring in children and young adults [1]. About 15–25% of Ewing's sarcoma (ES) patients will present with metastasis at diagnosis; however, those without detectable metastases frequently relapse after surgical resection due to the presence of micrometastases. Patients who present with metastases at diagnosis have a five-year survival rate of 25% [2]. Though multimodality treatment has improved survival in patients with localized disease, patients with metastatic or recurrent tumors have only limited benefit.

Standard treatment for localized disease includes surgery and chemotherapy with or without radiotherapy, depending on whether complete surgery is possible [3]. Multimodal cancer regimens have shown to increase the 5-year survival rate in patients with localized disease from <10% to >60%. The approach commonly used includes chemotherapy, followed by local surgery, with consolidation chemotherapy over the period of about a year. The most effective chemotherapeutic regimen has proved to include an alkylating agent (either ifosfamide or cyclophosphamide) plus doxorubicin [4]. After a 10-year followup of this regimen, it was found that 56% of patients remained free of disease whereas 42% of patients relapsed. Two patients in this study (0.6%) died from toxicity of the chemotherapy. Other agents commonly used include vincristine, dactinomycin, and etoposide.

In patients with metastatic disease, it is recommended that a similar chemotherapy regimen be administered, in addition to radiotherapy where appropriate [3]. Derivative studies have shown that using higher doses or time-compressed chemotherapy regimens does not confer any benefit to patients with metastatic disease [5]. The current multimodality treatments for those with metastatic disease have a 5-year survival of 20–40%. Patients who experience relapse fare poorly, with a 5-year survival of <20%. However, those who relapse more than two years after initial diagnosis have been shown to have a better outcome following retreatment. Regimens for patients who experience relapse of the disease commonly employ an alkylating agent and topoisomerase inhibitor, such as topotecan [3].

Ewing's sarcoma tumors are associated with unique chromosomal translocation between the EWS gene and the ETS transcription factor gene [6] of which more than 85% involve the EWS/FLI-1 fusion gene [7]. In the remaining ES cases, translocations involve other members of the ETS family, such as EWS-ERG [8]. It is largely accepted [9, 10] that the EWS/FLI-1 fusion gene product is involved in oncogenic properties of ESFT [11].

Although preclinical studies demonstrate oncogenic properties of EWS/FLI-1 in murine models, transfer of EWS/FLI-1 to normal human cells *in vitro* is not sufficient to transform to a malignant phenotype [12, 13]. This suggests that, in humans, additional genetic events other than the chromosomal translocation are required to cause tumorigenesis. Therefore, it is thought that several of the fusion's downstream gene targets also may play a part in the induced oncogenicity of EWS/FLI-1 in human cells [14]. Several genes, such as NKX2.2, GSTM4, and NR0B1, have in fact been found to enhance the oncogenicity of EWS/FLI-1 in normal cells [15–17]. EWS/FLI-1 protein has been shown to act as a transcriptional activator of tumorigenesis [18]. In particular, the EWS/FLI-1 protein expression is associated with activation of vascular endothelial growth factor (VEGF) and Caveolin-1 (CAV-1), which are known to contribute directly to tumor progression [19, 20].

Studies to define the effect of EWS/FLI-1 expression are limited due to lack of an appropriate model [14]. The cell of origin of ESFT is also of unknown origin. However, there is some evidence that Ewing's sarcoma cells originate from bone marrow-mesenchymal stem cells (MSCs) [21, 22]. Transfer of EWS/FLI-1 gene into murine bone marrow cells and murine mesenchymal cells results in small round cell phenotype tumors histologically similar to Ewing's sarcoma [23]. RNAi knockdown of EWS/FLI-1 in ES cell lines yields an MSC gene expression signaling profile [22]. However, attempts to express EWS/FLI-1 in mice or murine mesenchymal cells have resulted in the development of leukemia, not sarcoma, and no tumor formation whatsoever [24, 25].

## 2. RNA Interference

RNA interference is a natural process through which expression of a targeted gene is knocked down with high specificity and selectivity [26]. Independent of ribozymes, it was first used in 1998 in order to regulate muscle protein production in the nematode *C. elegans*. Since then, RNAi has been found to play a much larger role in the physiology of the human body and control of normal and malignant molecular signal pathways [27]. RNAi technology is also frequently used to study the function and signal effect of various genes in animal models [28].

Small interfering RNAs (siRNAs) are exogenous and work by regulating the degradation of the mRNA that is identical to the corresponding siRNA strand, resulting in the silencing of the respective genetic phenotype. The primary mechanism of siRNA is an RNase-H-like mRNA cleavage of the complementary mRNA sequence. After the mRNA is cleaved and released from its bound small RNA guide-strand, it is further degraded, while the RNA-induced silencing complex (RISC) moves on to cleave other mRNA [29]. In contrast, microRNAs (miRNAs) and short hairpin RNAs (shRNAs) are endogenous stem-loop structures that are, following RISC loading and processing, respectively noncomplementary or complementary (siRNA) to their cognate mRNA sequences [30]. Unlike siRNAs, which are not encoded by any genes, specific miRNA genes encode miRNAs as pri-miRNAs. miRNA effects mRNA degradation, p-body sequestration, and inhibition of translation [31]. shRNAs are processed as pre-miRNA in the nucleus of cells and utilize vectors introduced into cells with promoters to ensure continued expression of shRNA [32, 33]. Recently, we described a unique RNAi technology, called bifunctional shRNA (bi-shRNA), which is designed to concurrently induce target mRNA cleavage, mRNA degradation, p-body sequestration, and translational inhibition based on both RISC-loading cleavage dependent and independent mechanisms [34]. In all cases, however, the expression of the targeted gene has been shown to dramatically decrease, which in turn can indicate the physiological role of the gene product [35, 36].

## 3. siRNA

Mammalian dicer is an integral component of the RNA-interference pathway. Dicer processes pre-microRNA and double-strand RNA (dsRNA) to mature miRNA and siRNA, respectively, and transfers the processed products to the RISC [37, 38]. Dicer is a multidomain RNase III-related endonuclease responsible for processing dsRNA to siRNAs [39]. Dicer recognizes and preferentially binds to the terminal 2-nucleotide 3′ over-hang and cleaves dsRNAs into 21 to 22 nucleotide siRNAs [40, 41]. Dicer interacts with the double-stranded Tat-RNA-binding protein (TRBP) or PACT (PKR activating protein) to mediate RNA interference and miRNA processing. Knockdown of both TRBP and PACT in cultured cells leads to significant inhibition of gene silencing mediated by short hairpin RNA but not by siRNA, suggesting that TRBP and PACT function primarily at the step of siRNA production [42]. TRBP and PACT directly interact with each other and associate with Dicer to stimulate the cleavage of double-stranded or short hairpin RNA to siRNA [42]. Dicer knockout embryonic stem cells can effectively load processed siRNA onto RISC and carry out RNA interference as efficiently as Dicer^+^ embryonic stem cells [43]. So, it appears that in mammalian cells, a perfectly processed siRNA can be effectively loaded onto RISC for RNAi without the help of the TRBP/PACT/Dicer complex. The TRBP/PACT/Dicer complex, however, is required to process either conventional shRNA or long dsRNA to appropriate size and form for their loading onto RISC.

Duplex siRNA in association with holo-RISC, composed of at least Ago-2, Dicer, and TRBP, is identified as the RISC-loading complex (RLC) [44]. In the RLC, the two strands of the duplex are separated, resulting in the departure of the passenger strand [45–47]. The passenger strand is cleaved by the RNase-H-like activity of Ago-2, provided there are thermodynamically favorable conditions for passenger strand departure. This is referred to as the cleavage-dependent pathway [48]. There is also a cleavage-independent bypass pathway, in which the passenger strand with mismatches is induced to unwind and depart by an ATP-dependent helicase activity [45, 48, 49]. The RISC with single-stranded guide strand siRNA is then able to execute multiple rounds of RNA interference. ATP is not required for shRNA processing, RISC assembly, cleavage-dependent pathway, or multiple rounds of target-RNA cleavage [50–52]. Which of the duplex siRNA strands is incorporated into RISC is a matter of strand biasing favoring the strand with weakest base pair binding near the 5′ end [53].

Dynamically, siRNA steadily increases its accumulation in cells for four hours before plateau [54]. The cytoplasmic distribution of siRNA, delivered via TAT [41, 42, 44–52] peptide conjugation, appears to be in the perinuclear region forming a ring-like pattern around the nucleus [55] and has also been shown to accumulate rather evenly throughout the cytoplasm, although this may vary depending on the delivery mechanisms [56]. At 48 hours after injection, the majority of siRNA appears to have been degraded with only 1% fluorescence remaining in the cell. The spatial and temporal distribution of siRNA within the cell is in accord with the observed kinetics of siRNA-mediated RNA interference activity which peaks around 24 hours after delivery and diminishes within 48 hours. Though the siRNA method is an efficient means of silencing specific genetic products, there are a few limitations to *in vivo *use, including sensitivity to nucleases and a requirement for frequent dosing [57].

## 4. shRNA

shRNAs, as opposed to siRNAs, are synthesized in the nucleus of cells, being transcribed from plasmid or viral-based expression vectors, modified viruses, and extrachromosomal elements. Processing of shRNA is presumed to be very similar to the miRNA pathway, so miRNA studies have helped provided the basis for understanding shRNA synthesis. The primary transcripts are further processed and transported to the cytoplasm, and then incorporated into the RISC for activity [33]. shRNA can be transcribed by either RNA polymerase II or III through their respective promoters on the expression cassette. The resultant primary transcripts contain a hairpin-like stem-loop structure that is processed in the nucleus by a complex containing the RNase III enzyme Drosha and the double-stranded RNA binding domain protein DGCR8 [58]. The complex measures the hairpin and allows precise processing of the long primary transcripts into individual shRNAs with a 2 nt 3′ overhang [59]. The processed primary transcript is the pre-shRNA molecule. It is transported to the cytoplasm by Exportin 5 (and/or CRM1 for polymerase II), a Ran-GTP-dependent mechanism [60, 61]. In the cytoplasm the pre-shRNA is loaded onto another RNase III complex containing the RNase III enzyme Dicer and TRBP/PACT where the loop of the hairpin is processed off to form a double-stranded siRNA with 2 nt 3′ overhangs [62–64], although recent data support a primary role for Dicer recognition of the 5′ end in miRNA biogenesis [65]. The Dicer containing complex then coordinates loading onto the Ago2 protein containing RISC as described earlier for siRNA. Pre-shRNA has been found to be part of the RLC; thus, pre-shRNA may potentially directly associate with RLC rather than through a two steps process via a different Dicer/TRBP/PACT complex [66].

After loading onto RLC and passenger strand departure; both siRNA and shRNA in the RISC, in principle, should behave the same. There are, however, a number of differences between the two silencing methods. Due to its constant synthesis in host cells, shRNA offers more durable gene silencing than siRNA, which virtually disappears *in vivo* 48 hours after administration. Additionally, cost of manufacturing of exogenously delivered shRNA is markedly less than siRNA because siRNA requires frequent dosing in order to maximize its efficacy [57] which also has the potential of increasing off-target side effects [67]. The Argonaute family of proteins is the major component of RISC [68, 69]. Within the Argonaute family of proteins, only Ago2 contains target mRNA endonuclease activity necessary to cleave and release the passenger strand of the double-stranded stem [45, 46, 48]. Although AGO 1 has limited passenger strand endonuclease activity [70], the remaining three members of Argonaute family, Ago1, Ago3 and Ago4, which do not have identifiable target mRNA endonuclease activity, are also assembled into RISC. Thus, RISC loading can be further classified as cleavage dependent and cleavage independent [48].

The Argonaute family of proteins in RISCs are not only involved in the loading of siRNA or miRNA, but also implicated in both transcriptional (targeting heterochromatin) and posttranscriptional gene silencing. Ago protein complexes loaded with passenger strandless siRNA or miRNA seeks out complementary target sites in mRNAs, where endonucleolytically active Ago-2 cleaves mRNA to initiate mRNA degradation [71, 72]. Other Ago protein containing complexes without endonucleolytic activity predominantly bind to partially complementary target sites located at the 3′ UTR for translation repression through mRNA sequestration in processing bodies (p-bodies) [73–75]. The detailed mechanism of mRNA sequestration in p-bodies and later release from p-bodies is still a debated issue; deadenylation of the target mRNA which leads to destabilization of the mRNA was also observed to occur in p-bodies [76, 77]. Coimmunoprecipitation experiments which showed that RISCs are also strongly associated with polyribosomes or the small subunit ribosomes [66] and Ago-2 (actually identified as elF2c2), strongly suggest that RISC surveillance is compartmentalized with translational machinery of the cell. Details of the mechanism involving mRNA scanning and target mRNA identification are still largely unknown. Whatever the scanning or surveillance mechanism may be, once the target mRNA is identified, the target mRNA is either cleaved or conformationally changed following which both types of structures are routed to the p-body for either sequestration or degradation [76, 77]. The active siRNA or miRNA loaded complex is then released for additional rounds of gene silencing activity.

## 5. bi-shRNA

**Figure 1:**
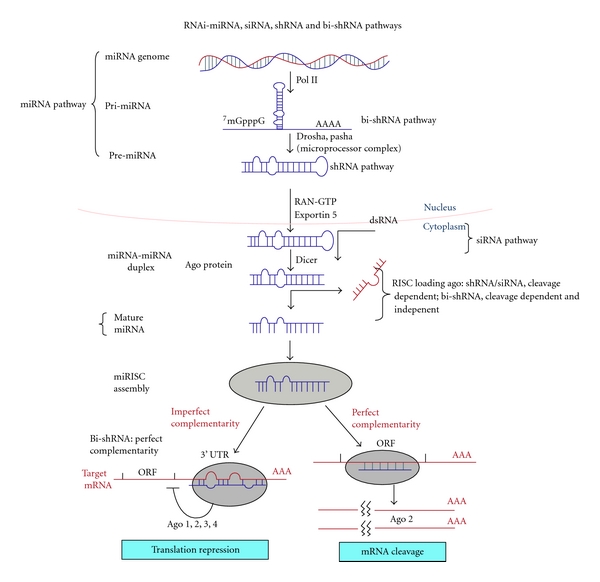
RNA interference mechanism comparing point of impact between shRNA, siRNA and bi-shRNA.

In utilizing the bifunctional technology, the RNAi structure is modified to take advantage of the endogenous gene silencing machinery, including both RISC loading pathways, to improve its efficiency and durability of action (see Figure 1). The two processing pathways primarily dependent on strand complementarity and/or access to RNase-H cleavage and, presumably, for final target effect, on interaction with Importin8 (Imp8) [78]. Simultaneous expression of both cleavage-dependent and -independent shRNAs (i.e., the bi-shRNA) in cells should achieve a higher level of efficacy, greater durability compared to siRNA, and a more rapid onset of gene expression silencing (the rate dependent on mRNA turnover and protein kinetics) compared to shRNA. Mechanistically, the “bifunctional” shRNA is able to simultaneously induce cleavage and non-RNase-H-mediated degradation of target mRNA, facilitate p-body sequestration, and also inhibit translation.

The design of the bi-shRNA expression unit is comprised of two stem-loop structures; one of them is composed of fully matched passenger and guide strands for cleavage-dependent RISC loading, the other is composed of a strategically placed passenger strand mismatch (at the position 9–12) for cleavage-independent RISC loading. These two shRNA structures are inserted in a miR-30 scaffold and are encoded by a plasmid vector [79]. The cleaved product siRNA is loaded onto Ago 2 containing RISC, while the cleavage-independent unit binds to Ago1–4 containing RISC. In contrast to miRNA, the cleavage-independent unit incorporates a guide strand complementary to its target mRNA. In summary, the enhanced effectiveness of the bi-shRNA has been shown to have greater durability and efficacy than other RNAi effectors due to its ability to induce RNase-H-like cleavage, to decap and deadenylate the target mRNA through noncleavage mediated processes, and also to inhibit translation.

There are several experimental observations that support this approach. In HEK293 cells transfected with tagged-Ago proteins, coimmunoprecipitation found similar sets of about 600 transcripts to be bound to Ago1, 2, 3, or 4 [66], suggesting that all four mammalian Ago protein containing RISCs are involved in RNAi function. Insofar as most mRNA have multiple miRNA target sites (with distance constraints) at their 3′ UTR, the miRNA-mediated RNAi system appears to be redundant for the targeted mRNAs allowing for cooperative downregulation to ensure target mRNA knockdown. The bifunctional shRNA approach mimics the natural process by mediating target mRNA knockdown through multiple RNAi pathways and complexes.

In *C. elegans,* structural features of small RNA precursors determine Argonaute loading [80]. Recently, Azuma-Mukai and coworkers observed overlapping association of miRNAs with hAgo-2 and hAgo-3; however, they presented evidence of limited discriminate loading onto hAgo-2 or hAgo-3 [81]. Further work is needed to resolve the specificity of miRNA loading onto different Ago containing RISCs. Although most miRNA target sites have been identified to be located at the 3′-UTR region, recent systemic identification of mRNAs recruited to hAgo-2 have identified additional mRNAs with target-sites located at the coding region and some at the 5′-UTR [82] albeit with the 3′-UTR persisting as the preferred target sequence site. Thus, hAgo-2 could initiate target mRNA degradation with its slicing activity in the coding region. Tay and colleagues recently found that many of the naturally occurring miRNA targets that are located in the coding region of embryonic regulated genes modulate embryonic stem cell differentiation [83], further supporting that miRNA can act through mRNA regions other than 3′-UTR.

## 6. Delivery Systems

Responses in a variety of cancers have been achieved via gene-specific RNAi in targeting putative or acknowledged oncogenes and/or presumptive dominant pathways. Using siRNA treatment, inhibition of proliferation, dysregulation of molecules involved in signal transduction, and increased chemosensitivity of malignancies have been demonstrated [84–86]. Although overexpressed nominated driver cancer genes are attractive targets for RNAi, many of these molecules are difficult to target with RNAi not because they are pharmacologically untargetable as is the case with small molecular inhibitors, but insofar as they are often essential to normal tissue homeostasis [87]. Therefore, it is important to find delivery systems that effectively knockdown gene targets in cancer cells while preserving normal, healthy cells. While viral vectors allow for highly efficient transgene expression, they are difficult to differentially target, can have limited intracellular cancer uptake as a result of downregulated or basal-lateral located viral receptors, can induce non-specific off-target immune/cytokine responses and, in some cases (e.g., retrovirus. AAV), integrate into the host's DNA [88]. Moreover, viral delivery systems, particularly AAV, although attractive from the standpoint of vector expression efficiency have unresolved limitations involving prevalence and induction of neutralizing antibodies [89], chromosome 19 and random DNA (preformed dsDNA strand breaks) [90] integration, germ line contamination [91], induced differentiation of human embryonic stem cells [92] and first trimester abortifaction [93, 94]. Recently, RNAi research has focused on the use of nonviral vectors, such as lipid-based carriers and polymers, as delivery systems. Nonviral systems have been shown to be safe and easier to mass-produce than viral vectors [95]. Although there continues to be difficulty in surmounting the obstacles of focused biodistribution and efficient transfection with systemically delivered nonviral vectors, research in this area continues to evolve with the proviso that the delivery system minimize the potential of off-target toxicity. A wide variety of potential vehicles have been and continue to be developed to address these issues. There are three major classes of nonviral delivery vehicle systems: synthetic polymers, natural/biodegradable polymers, and lipids; many of the vehicles that are showing promise are actually hybrids of these classes. For instance, there is a cyclodextrin-based cationic polymer which has been used successfully to deliver siRNA targeted to RRM2 in various *in vivo* cancer models [96, 97]. This preparation is currently in Phase I clinical trial. Lipid-based nanoparticles are showing shRNA and siRNA delivery potential [98]. Protiva Biotherapeutics and Alnylam have developed nanoparticles composed of a lipid-PEG conjugate that are capable of encapsulating and protecting nucleic acids for the purpose of systemic delivery. These stable nucleic acid lipid particles (SNALPs) were used in the first successful administration of siRNAs to a nonhuman primate [36, 99]. Silence Therapeutics has developed a lipid-based delivery vehicle specifically designed for siRNA delivery to endothelial cells. This vehicle, called AtuPLEX, is comprised of cationic and fusogenic lipids [100, 101]. This vehicle has been used effectively to knockdown protein kinase N3 in murine prostate and pancreatic cancer models, inhibiting cancer progression [102, 103]. More detailed discussions of delivery vehicles for shRNA [95, 104] and siRNA [105–108] as well as general discussions of organ and tissue-specific RNAi delivery may be found elsewhere [109–111].

## 7. Clinical Trials

The BCR-ABL fusion gene in chronic myeloid leukemia (described below) was the target of the first systemically administered siRNA drug in humans [112]. Although there were no adverse effects and an initial decrease of BCR-ABL was seen, further administrations of siRNA did not demonstrate continued effect. Several other promising trials are underway (Table 1 [112–117]), including one investigating the effect of siRNA on vascular endothelial growth factor (VEGF) kinesin spindle proteins (KSPs), which is nearing phase I completion [117, 118]. Preliminary results suggest reasonable safety and correlation of treatment with reduction in tumor vascular permeability as defined by DCE-MRI. A recently completed phase I trial of an autologous whole-cell vaccine expressing GM-CSF and incorporating *ex vivo* bi-shRNAi furin knockdown demonstrated significant reduction (>90% protein expression) of the endogenous immunosuppressors, TGF*β*_1_ and TGF*β*_2_ (the targets of the proprotein convertase furin) and suggested enhanced duration of survival compared to historical experience [116].

## 8. Knockdown Technologies to EWS/FLI-1

Antisense therapies, ribozymes, and RNAi have been used to silence EWS/FLI-1 in murine models and human cell lines [9, 119–123]. Studies have demonstrated fusion protein knockdown *in vitro* to correlate with a decreased tumor size and increased vulnerability of cells to apoptosis. Despite successful preclinical results, efforts to translate these therapies into the clinical arena do not exist. Improved delivery of RNAi molecules to tumor cells may enhance the effectiveness of targeted therapies *in vivo*. Additionally, EWS/FLI-1 lacks enzymatic function, making it difficult to identify its activity and discover specific EWS/FLI-1 inhibitors [124]. The small molecule inhibitor YK-4-279, which blocks the interaction of EWS/FLI-1 with RNA helicase A, was recently developed as the first molecule to directly target EWS/FLI-1 with clinical potential [125]. A potentially pertinent finding is that YK-4-279 effectively targets high ALDH activity Ewing's sarcoma stem cells [126].

It is believed that the most powerful mechanism of the fusion oncogene is its dysregulation of a number of downstream gene targets. As transfection of EWS/FLI-1 is unable to transform human mesenchymal progenitor cells (MPCs), focus has turned to exploration of the downstream products of EWS/FLI-1 as major cocontributors to oncogenicity [13]. Through these interactions, EWS/FLI-1 maintains a large degree of control in tumor development and progression, cell proliferation, and escape from apoptosis [127–129]. Consequently, a major strategy to impede cell transformation has been to identify each of these genes and develop integrated targeted therapies against them.

## 9. NKX2.2

NKX2.2, a target gene upregulated by EWS/FLI-1, acts as a transcriptional repressor in Ewing's sarcoma cells. However, this gene repression accounts for only a portion of the downregulation caused by the fusion gene product. The NKX2.2 gene has been shown to be critical for oncogenesis and the transformed phenotype of ES, making it an attractive target for gene therapy [129]. It was found, however, that EWS/FLI-1 did not upregulate NKX2.2 in murine ES cells, suggesting that the fusion uses different mechanisms depending on the cellular environment, and further, that NKX2.2 may not even play a role in murine ES cells [10]. Before therapies can be developed against this gene, it will be important to identify the exact mechanisms by which NKX2.2 transforms human ES cell lines. However, it has been difficult to effectively target transcription factors, so targeting a gene product of NKX2.2 that contributes to the oncogenicity of ES may be a more plausible, albeit indirect, option.

## 10. NR0B1

NR0B1 (DAX1), a nuclear hormone receptor and gene target of the fusion protein, has been known to act as a transcriptional corepressor and a context-dependent activator in ES cells, regulating genes due to its own upregulation by EWS/FLI-1 [130]. Expression of NR0B1 is critical for transformation and to maintain the phenotype of Ewing's sarcoma [16]. When EWS/FLI-1 was reduced using RNAi, NR0B1 transcription levels were subsequently reduced. Recently, it was discovered that NR0B1 and EWS/FLI-1 also physically interact during oncogenesis [130]. Several other nuclear hormone family members, such as estrogen receptor (although, unlike others, NR0B1 lacks a conventional DNA binding domain), have been isolated in a variety of cancers, and effective therapies have been developed due to an understanding of their role in tumorigenesis [131]. With further clarification of the NR0B1 gene's mechanisms, antagonists to modulate its activity and other targeted therapies could be developed as well.

## 11. GSTM4

Another direct target gene of EWS/FLI-1 is glutathione S-transferase M4 (GSTM4). Regulated through GGAA microsatellites, GSTM4 helps modulate resistance to chemotherapy in ES cells and is required for the ES phenotype [17]. Additionally, higher levels of GSTM4 have been found to correlate with a worse prognosis of ES patients. When RNAi was used to knockdown GSTM4 levels in patient-derived Ewing's sarcoma cell lines, an increase in sensitivity to the chemotherapeutic drug, etoposide, was seen [17]. Though no small molecule inhibitors for GSTM4 have been developed at this time, these findings suggest reducing levels of GSTM4 will have a beneficial effect on ES patients.

## 12. AURKA

EWS/FLI-1 is also known to upregulate Aurora kinase A (AURKA), a known transcriptional target of ES [132]. Additionally, AURKA is an important mitotic regulator, which supports its oncogenic transforming role in ES. This activity makes AURKA a viable target for the treatment of this disease. An AURKA inhibitor, MLN8054, was analyzed in a phase I clinical study in patients with advanced solid tumors, but no complete or partial responses were seen. However, another small molecule inhibitor, MLN8237, was recently shown to have success in reducing levels of the kinase in ESFT xenografts, as well as increasing sensitivity to apoptosis in an early phase clinical study [133].

## 13. EZH2

Members of the polycomb repressor group, comprised of the Polycomb repressor complexes PRC1 and PRC2/3, are often highly expressed in ES, and are known to play a large role in development and differentiation of cells [134]. In particular, EZH2 (the catalytic unit of the PRC2/3 complex), which represses gene expression involved in coordinating induction of tissue differentiation and maintains an undifferentiated, multipotent phenotype [135], is highly regulated by EWS/FLI-1 and is required for oncogenic transformation of Ewing's sarcoma cells [136]. Specifically, EZH2 was found to upregulate genes directly responsible for neuroectodermal and endothelial differentiation in Ewing's sarcoma cells. The large overexpression of EZH2 in ES cells may even be a result of direct mediation by EWS/FLI-1. Though EZH2 may be a novel target for therapy, the mechanisms by which EZH2 transforms ES cells are still unknown and will need to be understood before any inhibitor can be developed.

## 14. CAV1

Caveolin-1 (CAV1) is another direct gene target of EWS/FLI-1 that is overexpressed in ES cells. It is known to connect EWS/FLI-1 to a critical pathway which enables tumorigenesis in ES [14]. CAV1 also contains a GGAA microsatellite motif that EWS/FLI-1 binds to *in vivo* [137]. Additionally, it promotes tumor growth in mouse models, and is necessary for tumorigenesis in Ewing's sarcoma [20]. Recently, the gene target was shown to have some involvement in the chemoresistance of ES cells [138]. Because CAV1 exists in such high levels in ES, compared to the low amounts found in normal cells, it would be a suitable and efficient target for therapy. As of late, however, no gene silencing or small molecule inhibiting methods have been developed.

## 15. GLI1

EWS/FLI-1 is also known to upregulate GLI1, a commonly known oncogene with transcriptional activity. When GLI1 was inhibited in Ewing's sarcoma family tumor (ESFT) cell lines, the full ES phenotype was not seen [139]. In a subsequent study, it was shown that some GLI1 and EWS/FLI-1 transcriptional targets overlap [140]. Many hedgehog-GLIs (HH-GLIs) have been identified as EWS/FLI-1 targets, and it is thought that the fusion reaches these targets through its upregulation of GLI1, further implying GLI1's importance to ESFT. It would be beneficial to perform a shRNA knockdown of ES cells in order to identify other common targets of EWS/FLI-1 and GLI1, so that efficient targeted therapies could be developed. Currently, GANT58 and GANT61 small molecule modulators have been shown to inhibit the GLI1 pathway in Ewing's sarcoma, and GANT61 may even be effective *in vivo* [140]. Studies are currently underway investigating this claim [141].

Due to the lack of a good model system in Ewing's sarcoma, concerns have been raised about how well results from EWS/FLI-1 in murine cells will translate to human cells. Specifically, it has been shown that some of the gene targets mentioned, such as NKX2.2 and NR0B1, are not induced by the fusion in mouse cells, though they are prevalent in human cells [15, 16].

Suppression of EWS/FLI-1 fusion protein and decreased tumor growth was seen in ES cells* in vitro* and in murine models using antisense ODNs complementary to the fusion mRNA [120, 122]. Another study using mice showed decreased tumor size and levels of EWS/FLI-1 after the use of an antisense ODN nanocapsules [142]. In addition to successfully knocking down the fusion oncogene, this study offered a novel, nonviral vector for delivery of antisense ODNs.

In ES, as previously mentioned, siRNAs have been used to knockdown the expression of the EWS/FLI-1 fusion gene in SK-ES cell lines *in vitro* [9, 143]. This downregulation of EWS/FLI-1 was shown to significantly decrease proliferation of the treated cells and increase apoptosis in three-times as many cells as in the control cells. Additionally, siRNA knockdown effectively inhibited the metastatic nature of the SK-ES cell lines, suggesting that the presence of EWS/FLI-1 fusion protein is required for *in vitro* invasion. Another study showed knockdown of EWS/FLI-1 and suppression of tumor growth by systemic administration of siRNA in mice [119]. Most recently, siRNA-mediated sequence-specific suppression of EWS/FLI-1 inhibited proliferation of mouse ES xenografts *in vivo *[144].

Despite successful results in preclinical trials, effective tumor-specific systemic delivery of RNAi effectors has been elusive. Development of a targeted, nonimmunogenic RNAi delivery system will be required for efficient use of RNAi gene therapy in humans [119]. 

Efforts to knockdown the fusion oncogene in ES by shRNA in a 2009 study were also successful [145]. Not only did shRNA maintain a stable knockdown of EWS/FLI-1, but also a decrease in tumorigenicity of cells and tumor growth in mice was seen. Specifically, the shRNA treated cells resulted in smaller ES tumors, with a 40% decrease in size. This study also uncovered TOPK as a new target gene of EWS/FLI-1, which was effectively downregulated following the knockdown of the fusion gene. The downregulation of TOPK led to the decreased proliferation of the ES cells. Other studies of shRNA knockdown have likewise shown significant knockdown of the fusion gene, ultimately leading to attenuated oncogenicity and decreased proliferation of cancerous cells [10, 16, 146, 147]. More effective delivery vehicles, improving the ability of RNAi agents to selectively target tumor tissue and effectively navigate tumor cell entry, will be necessary for this therapeutic strategy to achieve clinical application.

## 16. Conclusion

The identification of the EWS/FLI-1 oncogenic fusion gene and demonstration of its broad-based expression in ES has broadened the potential for RNAi application to ES therapeusis [148]. An increasing number of RNAi studies provide a reasonable database to support the feasibility and effectiveness of EWS/FLI-1 knockdown, particularly when integrated with related downstream signals. Continued development of RNAi delivery methodology may permit more effective, low-morbidity gene silencing [149]. Translational applications are fast approaching. Our evolving understanding of the mechanisms through which EWS/FLI-1 protein and its interaction with downstream targets induces and supports the malignant phenotype further emphasizes the need for a therapeutic RNAi with multitarget potential [150].

## Figures and Tables

**Table 1:** RNAi drugs in human clinical trials [113–115].

Company	Product name	Disease	Target	Stage
Alnylam	ALN-RSV1	Respratory syncytial virus infection	Nucleocapsid (N) gene of RSV genome	Expanded phase II
Alnylam	ALN-VSP	Liver cancers and solid tumors	Kinesin spindle protein (KSP), VEGF	Phase I
Alnylam	ALN-TTR01	TTR-mediated amyloidosis	TTR	Phase I
Alnylam	ALN-PCS	hypercholesterolemia	PCSK9	Phase I
Benetec/City of Hope	—	AIDS lymphoma	rHIV7-shI-TAR-CCR5RZ	Phase I
Calando Pharmaceuticals	CALAA-01	Cancer and solid tumors	M2 subunit of ribonucleotide reductase (RRM2)	Phase I
Cequent Pharmaceuticals	CEQ508	FAP	*β*-Catenin	Phase I
Duke University	—	Metastatic melanoma	LMP2, LMP7, and MECL1	Phase I
OPKO Health	Bevasiranib	Wet age-related macular degeneration	VEGF	Expanded phase III
OPKO Health	Bevasiranib	Diabetic macular edema	VEGF	Phase II
Quark Pharmaceuticals	PF4523655/RTP801i14	Wet age-related macular degeneration	RTP801	Phase II
Quark Pharmaceuticals	PF4523655/RTP801i14	Diabetic macular edema	RTP801	Phase II
Quark Pharmaceuticals	QPI-1002/Akli5/I5NP	Acute kidney injury	P53	Phase I/IIa
Quark Pharmaceuticals	QPI-1002/DGFi	Delayed graft function in kidney transplantation	P53	Phase I/II
Sirna Therapeutics (MERCK)/Allergan	Sirna-027/AGN-745	Wet age-related macular degeneration	VEGFRI	Phase II
Silence Therapeutics	Atu027	Lung cancers	Protein kinase N3 (PKN3)	Phase I
SENETEK	—	Brain tumors glioblastomas	Tenascin-C	Phase I
Tekmira	ApoB SNALP	High LDL cholesterol	Apo B lipoprotein	Phase I/II
Tekmira	TKM-PLK1	Advanced solid tumor	PLK1	Phase I
TransDerm, Inc	TD101	Pachyonichia congenita	Keratin 6a (K6a)	Phase I
University of Duisbur-Essen	—	Chronic myeloid leukaemia	*bcr-abl*	Single patient
Gradalis, Inc.	FANG	Advanced cancer	Furin	Phase I
Gradalis, Inc.	FANG	Ovarian	Furin	Phase II
Gradalis, Inc.	FANG	Melanoma	Furin	Phase II
Gradalis, Inc.	FANG	Colon cancer	Furin	Phase II

## References

[1] Arndt CAS, Crist WM (1999). Common musculoskeletal tumors of childhood and adolescence. *The New England Journal of Medicine*.

[2] Linabery AM, Ross JA (2008). Childhood and adolescent cancer survival in the US by race and ethnicity for the diagnostic period 1975–1999. *Cancer*.

[3] Paulussen M, Bielack S, Jürgens H, Casali PG (2009). Ewing's sarcoma of the bone: ESMO clinical recommendations for diagnosis, treatment and follow-up. *Annals of Oncology*.

[4] Bacci G, Ferrari S, Bertoni F (2000). Prognostic factors in nonmetastatic Ewing’s sarcoma of bone treated with adjuvant chemotherapy: analysis of 359 patients at the Istituto Ortopedico Rizzoli. *Journal of Clinical Oncology*.

[5] Pinkerton CR, Bataillard A, Guillo S, Oberlin O, Fervers B, Philip T (2001). Treatment strategies for metastatic Ewing’s sarcoma. *European Journal of Cancer*.

[6] Kovar H, Aryee D, Zoubek A (1999). The Ewing family of tumors and the search for the Achilles’ heel. *Current Opinion in Oncology*.

[7] Bennicelli JL, Barr FG (2002). Chromosomal translocations and sarcomas. *Current Opinion in Oncology*.

[8] Peter M, Couturier J, Pacquement H (1997). A new member of the ETS family fused to EWS in Ewing tumors. *Oncogene*.

[9] Chansky HA, Barahmand-pour F, Mei Q (2004). Targeting of EWS/FLI-1 by RNA interference attenuates the tumor phenotype of Ewing’s sarcoma cells in vitro. *Journal of Orthopaedic Research*.

[10] Owen LA, Lessnick SL (2006). Identification of target genes in their native cellular context: an analysis of EWS/FLI in Ewing’s sarcoma. *Cell Cycle*.

[11] Mackintosh C, Madoz-Gúrpide J, Ordóñez JL, Osuna D, Herrero-Martín D (2010). The molecular pathogenesis of Ewing’s sarcoma. *Cancer Biology and Therapy*.

[12] Braunreiter CL, Hancock JD, Coffin CM, Boucher KM, Lessnick SL (2006). Expression of EWS-ETS fusions in NIH3T3 cells reveals significant differences to Ewing’s sarcoma. *Cell Cycle*.

[13] Riggi N, Suvà ML, Suvà D (2008). EWS-FLI-1 expression triggers a ewing’s sarcoma initiation program in primary human mesenchymal stem cells. *Cancer Research*.

[14] Toomey EC, Schiffman JD, Lessnick SL (2010). Recent advances in the molecular pathogenesis of Ewing’s sarcoma. *Oncogene*.

[15] Owen LA, Kowalewski AA, Lessnick SL (2008). EWS/FLI mediates transcriptional repression via NKX2.2 during oncogenic transformation in Ewing’s sarcoma. *PLoS One*.

[16] Kinsey M, Smith R, Lessnick SL (2006). NR0B1 is required for the oncogenic phenotype mediated by EWS/FLI in Ewing’s sarcoma. *Molecular Cancer Research*.

[17] Luo W, Gangwal K, Sankar S, Boucher KM, Thomas D, Lessnick SL (2009). GSTM4 is a microsatellite-containing EWS/FLI target involved in Ewing’s sarcoma oncogenesis and therapeutic resistance. *Oncogene*.

[18] Prieur A, Tirode F, Cohen P, Delattre O (2004). EWS/FLI-1 silencing and gene profiling of Ewing cells reveal downstream oncogenic pathways and a crucial role for repression of insulin-like growth factor binding protein 3. *Molecular and Cellular Biology*.

[19] Fuchs B, Inwards CY, Janknecht R (2004). Vascular endothelial growth factor expression is up-regulated by ews-ets oncoproteins and sp1 and may represent an independent predictor of survival in Ewing’s sarcoma. *Clinical Cancer Research*.

[20] Tirado OM, Mateo-Lozano S, Villar J (2006). Caveolin-1 (CAV1) is a target of EWS/FLI-1 and a key determinant of the oncogenic phenotype and tumorigenicity of Ewing’s sarcoma cells. *Cancer Research*.

[21] Riggi N, Cironi L, Provero P (2005). Development of Ewing’s sarcoma from primary bone marrow-derived mesenchymal progenitor cells. *Cancer Research*.

[22] Tirode F, Laud-Duval K, Prieur A, Delorme B, Charbord P, Delattre O (2007). Mesenchymal stem cell features of ewing tumors. *Cancer Cell*.

[23] Castillero-Trejo Y, Eliazer S, Xiang L, Richardson JA, Ilaria RL (2005). Expression of the EWS/FLI-1 oncogene in murine primary bone-derived cells results in EWS/FLI-1-dependent, Ewing sarcoma-like tumors. *Cancer Research*.

[24] Torchia EC, Boyd K, Rehg JE, Qu C, Baker SJ (2007). EWS/FLI-1 induces rapid onset of myeloid/erythroid leukemia in mice. *Molecular and Cellular Biology*.

[25] Lin PP, Pandey MK, Jin F (2008). EWS-FLI1 induces developmental abnormalities and accelerates sarcoma formation in a transgenic mouse model. *Cancer Research*.

[26] Fire A, Xu S, Montgomery MK, Kostas SA, Driver SE, Mello CC (1998). Potent and specific genetic interference by double-stranded RNA in caenorhabditis elegans. *Nature*.

[27] Carthew RW, Sontheimer EJ (2009). Origins and Mechanisms of miRNAs and siRNAs. *Cell*.

[28] Martin SE, Caplen NJ (2007). Applications of RNA interference in mammalian systems. *Annual Review of Genomics and Human Genetics*.

[29] Elbashir SM, Harborth J, Lendeckel W, Yalcin A, Weber K, Tuschl T (2001). Duplexes of 21-nucleotide RNAs mediate RNA interference in cultured mammalian cells. *Nature*.

[30] Lewis BP, Shih IH, Jones-Rhoades MW, Bartel DP, Burge CB (2003). Prediction of mammalian MicroRNA targets. *Cell*.

[31] Teixeira D, Sheth U, Valencia-Sanchez MA, Brengues M, Parker R (2005). Processing bodies require RNA for assembly and contain nontranslating mRNAs. *RNA*.

[32] Thomas CE, Ehrhardt A, Kay MA (2003). Progress and problems with the use of viral vectors for gene therapy. *Nature Reviews Genetics*.

[33] Cullen BR (2005). RNAi the natural way. *Nature Genetics*.

[34] Rao DD, Maples PB, Senzer N (2010). Enhanced target gene knockdown by a bifunctional shRNA: a novel approach of RNA interference. *Cancer Gene Therapy*.

[35] Soutschek J, Akinc A, Bramlage B (2004). Therapeutic silencing of an endogenous gene by systemic administration of modified siRNAs. *Nature*.

[36] Zimmermann TS, Lee ACH, Akinc A (2006). RNAi-mediated gene silencing in non-human primates. *Nature*.

[37] Bernstein E, Caudy AA, Hammond SM, Hannon GJ (2001). Role for a bidentate ribonuclease in the initiation step of RNA interference. *Nature*.

[38] Provost P, Dishart D, Doucet J, Frendewey D, Samuelsson B, Rådmark O (2002). Ribonuclease activity and RNA binding of recombinant human Dicer. *The EMBO Journal*.

[39] Hammond SM, Caudy AA, Hannon GJ (2001). Post-transcriptional gene silencing by double-stranded RNA. *Nature Reviews Genetics*.

[40] Carmell MA, Hannon GJ (2004). RNase III enzymes and the initiation of gene silencing. *Nature Structural and Molecular Biology*.

[41] MacRae IJ, Zhou K, Li F (2006). Structural basis for double-stranded RNA processing by Dicer. *Science*.

[42] Kok KH, Ng MHJ, Ching YP, Jin DY (2007). Human TRBP and PACT directly interact with each other and associate with dicer to facilitate the production of small interfering RNA. *The Journal of Biological Chemistry*.

[43] Murchison EP, Partridge JF, Tam OH, Cheloufi S, Hannon GJ (2005). Characterization of Dicer-deficient murine embryonic stem cells. *Proceedings of the National Academy of Sciences of the United States of America*.

[44] Robb GB, Rana TM (2007). Rna helicase a interacts with risc in human cells and functions in risc loading. *Molecular Cell*.

[45] Matranga C, Tomari Y, Shin C, Bartel DP, Zamore PD (2005). Passenger-strand cleavage facilitates assembly of siRNA into Ago2-containing RNAi enzyme complexes. *Cell*.

[46] Rand TA, Petersen S, Du F, Wang X (2005). Argonaute2 cleaves the anti-guide strand of siRNA during RISC activation. *Cell*.

[47] Leuschner PJF, Ameres SL, Kueng S, Martinez J (2006). Cleavage of the siRNA passenger strand during RISC assembly in human cells. *EMBO Reports*.

[48] Preall JB, Sontheimer EJ (2005). RNAi: RISC gets loaded. *Cell*.

[49] Haley B, Zamore PD (2004). Kinetic analysis of the RNAi enzyme complex. *Nature Structural and Molecular Biology*.

[50] Gregory RI, Chendrimada TP, Cooch N, Shiekhattar R (2005). Human RISC couples microRNA biogenesis and posttranscriptional gene silencing. *Cell*.

[51] Maniataki E, Mourelatos Z (2005). A human, ATP-independent, RISC assembly machine fueled by pre-miRNA. *Genes and Development*.

[52] Collins RE, Cheng X (2006). Structural and biochemical advances in mammalian RNAi. *Journal of Cellular Biochemistry*.

[53] Boudreau RL, Monteys AM, Davidson BL (2008). Minimizing variables among hairpin-based RNAi vectors reveals the potency of shRNAs. *RNA*.

[54] Grünweller A, Gillen C, Erdmann VA, Kurreck J (2003). Cellular uptake and localization of a cy3-labeled sirna specific for the serine/threonine kinase pim-1. *Oligonucleotides*.

[55] Chiu YL, Ali A, Chu CY, Cao H, Rana TM (2004). Visualizing a correlation between siRNA localization, cellular uptake, and RNAi in living cells. *Chemistry and Biology*.

[56] Ohrt T, Merkle D, Birkenfeld K, Echeverri CJ, Schwille P (2006). In situ fluorescence analysis demonstrates active siRNA exclusion from the nucleus by Exportin 5. *Nucleic Acids Research*.

[57] Wang Z, Rao DD, Senzer N, Nemunaitis J (2011). RNA interference and cancer therapy. *Pharmaceutical Research*.

[58] Lee Y, Ahn C, Han J (2003). The nuclear RNase III Drosha initiates microRNA processing. *Nature*.

[59] Zhang H, Kolb FA, Brondani V, Billy E, Filipowicz W (2002). Human Dicer preferentially cleaves dsRNAs at their termini without a requirement for ATP. *The EMBO Journal*.

[60] Lee Y, Jeon K, Lee JT, Kim S, Kim VN (2002). MicroRNA maturation: stepwise processing and subcellular localization. *The EMBO Journal*.

[61] Cullen BR (2004). Transcription and processing of human microRNA precursors. *Molecular Cell*.

[62] Yi R, Qin Y, Macara IG, Cullen BR (2003). Exportin-5 mediates the nuclear export of pre-microRNAs and short hairpin RNAs. *Genes and Development*.

[63] Lund E, Güttinger S, Calado A, Dahlberg JE, Kutay U (2004). Nuclear export of microrna precursors. *Science*.

[64] Lee YS, Nakahara K, Pham JW (2004). Distinct roles for Drosophila Dicer-1 and Dicer-2 in the siRNA/miRNA silencing pathways. *Cell*.

[65] Park J-E, Heo I, Tian Y (2011). Dicer recognizes the 5′ end of RNA for efficient and accurate processing. *Nature*.

[66] Landthaler M, Gaidatzis D, Rothballer A (2008). Molecular characterization of human Argonaute-containing ribonucleoprotein complexes and their bound target mRNAs. *RNA*.

[67] Rao DD, Senzer N, Cleary MA, Nemunaitis J (2009). Comparative assessment of siRNA and shRNA off target effects: what is slowing clinical development. *Cancer Gene Therapy*.

[68] Hammond SM, Boettcher S, Caudy AA, Kobayashi R, Hannon GJ (2001). Argonaute2, a link between genetic and biochemical analyses of RNAi. *Science*.

[69] Sontheimer EJ, Carthew RW (2004). Argonaute journeys into the heart of RISC. *Science*.

[70] Wang B, Li S, Qi HH, Chowdhury D, Shi Y, Novina CD (2009). Distinct passenger strand and mRNA cleavage activities of human Argonaute proteins. *Nature Structural and Molecular Biology*.

[71] Hutvágner G, Zamore PD (2002). A microRNA in a multiple-turnover RNAi enzyme complex. *Science*.

[72] Yekta S, Shih IH, Bartel DP (2004). MicroRNA-directed cleavage of HOXB8 mRNA. *Science*.

[73] Humphreys DT, Westman BJ, Martin DIK, Preiss T (2005). MicroRNAs control translation initiation by inhibiting eukaryotic initiation factor 4E/cap and poly(A) tail function. *Proceedings of the National Academy of Sciences of the United States of America*.

[74] Pillai RS, Bhattacharyya SN, Artus CG (2005). Molecular biology: inhibition of translational initiation by let-7 microRNA in human cells. *Science*.

[75] Thermann R, Hentze MW (2007). Drosophila miR2 induces pseudo-polysomes and inhibits translation initiation. *Nature*.

[76] Valencia-Sanchez MA, Liu J, Hannon GJ, Parker R (2006). Control of translation and mRNA degradation by miRNAs and siRNAs. *Genes and Development*.

[77] Parker R, Sheth U (2007). P Bodies and the control of mRNA translation and degradation. *Molecular Cell*.

[78] Weinmann L, Höck J, Ivacevic T (2009). Importin 8 is a gene silencing factor that targets argonaute proteins to distinct mRNAs. *Cell*.

[79] Rao DD, Vorhies JS, Senzer N, Nemunaitis J (2009). siRNA vs. shRNA: similarities and differences. *Advanced Drug Delivery Reviews*.

[80] Steiner FA, Hoogstrate SW, Okihara KL (2007). Structural features of small RNA precursors determine Argonaute loading in Caenorhabditis elegans. *Nature Structural and Molecular Biology*.

[81] Azuma-Mukai A, Oguri H, Mituyama T (2008). Characterization of endogenous human Argonautes and their miRNA partners in RNA silencing. *Proceedings of the National Academy of Sciences of the United States of America*.

[82] Hendrickson DG, Hogan DJ, Herschlag D, Ferrell JE, Brown PO (2008). Systematic identification of mrNAs recruited to argonaute 2 by specific microRNAs and corresponding changes in transcript abundance. *PLoS One*.

[83] Tay Y, Zhang J, Thomson AM, Lim B, Rigoutsos I (2008). MicroRNAs to *Nanog, Oct4* and *Sox2* coding regions modulate embryonic stem cell differentiation. *Nature*.

[84] Sonoke S, Ueda T, Fujiwara K (2008). Tumor regression in mice by delivery of Bcl-2 small interfering RNA with pegylated cationic liposomes. *Cancer Research*.

[85] Fan Y, Zhang YL, Wu Y (2008). Inhibition of signal transducer and activator of transcription 3 expression by RNA interference suppresses invasion through inducing anoikis in human colon cancer cells. *World Journal of Gastroenterology*.

[86] Saad M, Garbuzenko OB, Minko T (2008). Co-delivery of siRNA and an anticancer drug for treatment of multidrug-resistant cancer. *Nanomedicine*.

[87] Ashihara E, Kawata E, Maekawa T (2010). Future prospect of RNA interference for cancer therapies. *Current Drug Targets*.

[88] Hacein-Bey-Abina S, Von Kalle C, Schmidt M (2003). A serious adverse event after successful gene therapy for X-linked severe combined immunodeficiency. *The New England Journal of Medicine*.

[89] Boutin S, Monteilhet V, Veron P (2010). Prevalence of serum IgG and neutralizing factors against adeno-associated virus (AAV) types 1, 2, 5, 6, 8, and 9 in the healthy population: implications for gene therapy using AAV vectors. *Human Gene Therapy*.

[90] Henckaerts E, Linden RM (2010). Adeno-associated virus: a key to the human genome?. *Future Virology*.

[91] High KA (2003). Theodore E. Woodward Award. AAV-mediated gene transfer for hemophilia. *Transactions of the American Clinical and Climatological Association*.

[92] Botquin V, Cid-Arregui A, Schlehofer JR (1994). Adeno-associated virus type 2 interferes with early development of mouse embryos. *Journal of General Virology*.

[93] Tobiasch E, Rabreau M, Geletneky K (1994). Detection of adeno-associated virus DNA in human genital tissue and in material from spontaneous abortion. *Journal of Medical Virology*.

[94] Malhomme O, Dutheil N, Rabreau M, Armbruster-Moraes E, Schlehofer JR, Dupressoir T (1997). Human genital tissues containing DNA of adeno-associated virus lack DNA sequences of the helper viruses adenovirus, herpes simplex virus or cytomegalovirus but frequently contain human papillomavirus DNA. *Journal of General Virology*.

[95] Vorhies JS, Nemunaitis J (2007). Nonviral delivery vehicles for use in short hairpin RNA-based cancer therapies. *Expert Review of Anticancer Therapy*.

[96] Heidel JD (2006). Linear cyclodextrin-containing polymers and their use as delivery agents. *Expert Opinion on Drug Delivery*.

[97] Heidel JD, Liu JYC, Yen Y (2007). Potent siRNA inhibitors of ribonucleotide reductase subunit RRM2 reduce cell proliferation in vitro and in vivo. *Clinical Cancer Research*.

[98] Moreira JN, Santos A, Moura V, De Uma MCP, Simões S (2008). Non-viral lipid-based nanoparticles for targeted cancer systemic gene silencing. *Journal of Nanoscience and Nanotechnology*.

[99] Blow N (2007). Small RNAs: delivering the future. *Nature*.

[100] Santel A, Aleku M, Keil O (2006). RNA interference in the mouse vascular endothelium by systemic administration of siRNA-lipoplexes for cancer therapy. *Gene Therapy*.

[101] Santel A, Aleku M, Keil O (2006). A novel siRNA-lipoplex technology for RNA interference in the mouse vascular endothelium. *Gene Therapy*.

[102] Aleku M, Fisch G, Möpert K (2008). Intracellular localization of lipoplexed siRNA in vascular endothelial cells of different mouse tissues. *Microvascular Research*.

[103] Aleku M, Schulz P, Keil O (2008). Atu027, a liposomal small interfering RNA formulation targeting protein kinase N3, inhibits cancer progression. *Cancer Research*.

[104] Vorhies JS, Nemunaitis JJ (2009). Synthetic vs. natural/biodegradable polymers for delivery of shRNA-based Cancer Therapies. *Methods in Molecular Biology*.

[105] Kumar P, Ban HS, Kim SS (2008). T cell-specific siRNA delivery suppresses HIV-1 infection in humanized mice. *Cell*.

[106] Gao K, Huang L (2009). Nonviral methods for siRNA delivery. *Molecular Pharmaceutics*.

[107] Aigner A (2007). Nonviral in vivo delivery of therapeutic small interfering RNAs. *Current Opinion in Molecular Therapeutics*.

[108] Akinc A, Zumbuehl A, Goldberg M (2008). A combinatorial library of lipid-like materials for delivery of RNAi therapeutics. *Nature Biotechnology*.

[109] Ghatak S, Hascall V, Berger F (2008). Tissue-specific shRNA delivery: a novel approach for gene therapy in cancer. *Connective Tissue Research*.

[110] Nguyen T, Menocal EM, Harborth J, Fruehauf JH (2008). RNAi therapeutics: an update on delivery. *Current Opinion in Molecular Therapeutics*.

[111] Kumar P, Wu H, McBride JL (2007). Transvascular delivery of small interfering RNA to the central nervous system. *Nature*.

[112] Koldehoff M, Steckel NK, Beelen DW, Elmaagacli AH (2007). Therapeutic application of small interfering RNA directed against bcr-abl transcripts to a patient with imatinib-resistant chronic myeloid leukaemia. *Clinical and Experimental Medicine*.

[113] López-Fraga M, Martínez T, Jiménez A (2009). RNA interference technologies and therapeutics: from basic research to products. *BioDrugs*.

[114] Tiemann K, Rossi JJ (2009). RNAi-based therapeutics-current status, challenges and prospects. *EMBO Molecular Medicine*.

[115] Vaishnaw AK, Gollob J, Gamba-Vitalo C (2010). A status report on RNAi therapeutics. *Silence*.

[116] Senzer N, Barve M, Kuhn J (2012). Phase I trial of “bi-shRNAi^furin^/GMCSF DNA/autologous tumor cell” vaccine (FANG) in advanced cancer. *Molecular Therapy*.

[117] http://www.medicalnewstoday.com/releases/145218.php.

[118] Judge AD, Robbins M, Tavakoli I (2009). Confirming the RNAi-mediated mechanism of action of siRNA-based cancer therapeutics in mice. *The Journal of Clinical Investigation*.

[119] Hu-Lieskovan S, Heidel JD, Bartlett DW, Davis ME, Triche TJ (2005). Sequence-specific knockdown of EWS-FLI1 by targeted, nonviral delivery of small interfering RNA inhibits tumor growth in a murine model of metastatic Ewing’s sarcoma. *Cancer Research*.

[120] Maksimenko A, Lambert G, Bertrand JR, Fattal E, Couvreur P, Malvy C (2003). Therapeutic potentialities of ews-fli-1 mRNA-targeted vectorized antisense oligonucleotides. *Annals of the New York Academy of Sciences*.

[121] Ouchida M, Ohno T, Fujimura Y, Rao VN, Reddy ESP (1995). Loss of tumorigenicity of Ewing’s sarcoma cells expressing antisense RNA to EWS-fusion transcripts. *Oncogene*.

[122] Tanaka K, Iwakuma T, Harimaya K, Sato H, Iwamoto Y (1997). EWS-Fli1 antisense oligodeoxynucleotide inhibits proliferation of human Ewing’s sarcoma and primitive neuroectodermal tumor cells. *The Journal of Clinical Investigation*.

[123] Toretsky JA, Connell Y, Neckers L, Bhat NK (1997). Inhibition of EWS-FLI-1 fusion protein with antisense oligodeoxynucleotides. *Journal of Neuro-Oncology*.

[124] Erkizan HV, Uversky VN, Toretsky JA (2010). Oncogenic partnerships: EWS-FLI1 protein interactions initiate key pathways of Ewing’s sarcoma. *Clinical Cancer Research*.

[125] Erkizan HV, Kong Y, Merchant M (2009). A small molecule blocking oncogenic protein EWS-FLI1 interaction with RNA helicase A inhibits growth of Ewing’s sarcoma. *Nature Medicine*.

[126] Awad O, Yustein JT, Shah P (2010). High ALDH activity identifies chemotherapy-resistant Ewing’s sarcoma stem cells that retain sensitivity to EWS-Fli1 inhibition. *PLoS One*.

[127] Scotlandi K, Benini S, Nanni P (1998). Blockage of insulin-like growth factor-I receptor inhibits the growth of Ewing’s sarcoma in athymic mice. *Cancer Research*.

[128] Hahm KB, Cho K, Lee C (1999). Repression of the gene encoding the TGF-*β* type II receptor is a major target of the EWS-FLI1 oncoprotein. *Nature Genetics*.

[129] Smith R, Owen LA, Trem DJ (2006). Expression profiling of EWS/FLI identifies NKX2.2 as a critical target gene in Ewing’s sarcoma. *Cancer Cell*.

[130] Kinsey M, Smith R, Iyer AK, McCabe ERB, Lessnick SL (2009). EWS/FLI and its downstream target NR0B1 interact directly to modulate transcription and oncogenesis in Ewing’s sarcoma. *Cancer Research*.

[131] Baselga J, Norton L (2002). Focus on breast cancer. *Cancer Cell*.

[132] Wakahara K, Ohno T, Kimura M (2008). EWS-Fli1 up-regulates expression of the Aurora A and Aurora B kinases. *Molecular Cancer Research*.

[133] Maris JM, Morton CL, Gorlick R (2010). Initial testing of the Aurora kinase a inhibitor MLN8237 by the Pediatric Preclinical Testing Program (PPTP). *Pediatric Blood and Cancer*.

[134] Bracken AP, Dietrich N, Pasini D, Hansen KH, Helin K (2006). Genome-wide mapping of polycomb target genes unravels their roles in cell fate transitions. *Genes and Development*.

[135] Ciarapica R, Miele L, Giordano A, Locatelli F, Rota R (2011). Enhancer of zeste homolog 2 (EZH2) in pediatric soft tissue sarcomas: first implications. *BMC Medicine*.

[136] Richter GHS, Plehm S, Fasan A (2009). EZH2 is a mediator of EWS/FLI1 driven tumor growth and metastasis blocking endothelial and neuro-ectodermal differentiation. *Proceedings of the National Academy of Sciences of the United States of America*.

[137] Gangwal K, Sankar S, Hollenhorst PC (2008). Microsatellites as EWS/FLI response elements in Ewing’s sarcoma. *Proceedings of the National Academy of Sciences of the United States of America*.

[138] Tirado OM, MacCarthy CM, Fatima N, Villar J, Mateo-Lozano S, Notario V (2010). Caveolin-1 promotes resistance to chemotherapy-induced apoptosis in Ewing’s sarcoma cells by modulating PKC*α* phosphorylation. *International Journal of Cancer*.

[139] Zwerner JP, Joo J, Warner KL (2008). The EWS/FLI1 oncogenic transcription factor deregulates GLI1. *Oncogene*.

[140] Joo J, Christensen L, Warner K (2009). GLI1 is a central mediator of EWS/FLI1 signaling in Ewing Tumors. *PLoS One*.

[141] http://www.curesarcoma.org/index.php/research_grants/past_grantees/2010_sfa_research_grant_recipients/.

[142] Lambert G, Bertrand JR, Fattal E (2000). EWS Fli-1 antisense nanocapsules inhibits Ewing sarcoma-related tumor in mice. *Biochemical and Biophysical Research Communications*.

[143] Dohjima T, Lee NS, Li H, Ohno T, Rossi JJ (2003). Small interfering RNAs expressed from a Pol III promoter suppress the EWS/Fli-1 transcript in an Ewing sarcoma cell line. *Molecular Therapy*.

[144] Takigami I, Ohno T, Kitade Y (2011). Synthetic siRNA targeting the breakpoint of EWS/Fli-1 inhibits growth of Ewing sarcoma xenografts in a mouse model. *International Journal of Cancer*.

[145] Herrero-Martín D, Osuna D, Ordó˜ez JL (2009). Stable interference of EWS-FLI1 in an Ewing sarcoma cell line impairs IGF-1/IGF-1R signalling and reveals TOPK as a new target. *British Journal of Cancer*.

[146] Carrillo J, García-Aragoncillo E, Azorín D (2007). Cholecystokinin down-regulation by RNA interference impairs Ewing tumor growth. *Clinical Cancer Research*.

[147] Stegmaier K, Wong JS, Ross KN (2007). Signature-based small molecule screening identifies cytosine arabinoside as an EWS/FLI modulator in ewing sarcoma. *PLoS Medicine*.

[148] Damm-Welk C, Fuchs U, Wössmann W, Borkhardt A (2003). Targeting oncogenic fusion genes in leukemias and lymphomas by RNA interference. *Seminars in Cancer Biology*.

[149] Shi Q, Nguyen AT, Angell Y (2010). A combinatorial approach for targeted delivery using small molecules and reversible masking to bypass nonspecific uptake in vivo. *Gene Therapy*.

[150] Tong AW, Jay CM, Senzer N, Maples PB, Nemunaitis J (2009). Systemic therapeutic gene delivery for cancer: crafting Paris’ arrow. *Current Gene Therapy*.

